# MicroRNA-153 Decreases Tryptophan Catabolism and Inhibits Angiogenesis in Bladder Cancer by Targeting Indoleamine 2,3-Dioxygenase 1

**DOI:** 10.3389/fonc.2019.00619

**Published:** 2019-07-10

**Authors:** Wentao Zhang, Shiyu Mao, Donghui Shi, Junfeng Zhang, Ziwei Zhang, Yadong Guo, Yuan Wu, Ruiliang Wang, Longsheng Wang, Yong Huang, Xudong Yao

**Affiliations:** ^1^Department of Urology, Shanghai Tenth People's Hospital, Tongji University, Shanghai, China; ^2^Anhui Medical University, Shanghai Clinical College, Hefei, China; ^3^Department of Urology, Suzhou Wuzhong People's Hospital, Wuzhong, China; ^4^Department of Urology, The First Affiliated Hospital of Inner Mongolia Medical University, Hohhot, China

**Keywords:** bladder cancer, miR-153, tryptophan catabolism, angiogenesis, indoleamine 2, 3-dioxygenase 1

## Abstract

**Background:** Metastasis is the primary cause of cancer deaths, warranting further investigation. This study assessed microRNA-153 (miR-153) expression in bladder cancer tissues and investigated the underlying molecular mechanism of miR-153-mediated regulation of bladder cancer cells.

**Methods:** Paired tissue specimens from 45 bladder cancer patients were collected for qRT-PCR. The Cancer Genome Atlas (TCGA) dataset was used to identify associations of miR-153 with bladder cancer prognosis. Bladder cancer tissues and immortalized cell lines were used for the following experiments: miR-153 mimics and indoleamine 2,3-dioxygenase 1 (IDO1) siRNA transfection; Western blot, cell viability, colony formation, and Transwell analyses; nude mouse xenograft; and chicken embryo chorioallantoic membrane angiogenesis (CAM) assays. Human umbilical vein endothelial cells (HUVECs) were co-cultured with bladder cancer cells for the tube formation assay. The luciferase reporter assay was used to confirm miR-153-targeting genes.

**Results:** miR-153 expression was downregulated in bladder cancer tissues and cell lines, and reduced miR-153 expression was associated with advanced tumor stage and poor overall survival of patients. Moreover, miR-153 expression inhibited bladder cancer cell growth by promoting tumor cell apoptosis, migration, invasion, and endothelial mesenchymal transition (EMT) *in vitro* and tumor xenograft growth *in vivo*, while miR-153 expression suppressed HUVEC and CAM angiogenesis. At the gene level, miR-153 targeted IDO1 expression and inhibited bladder cancer cell tryptophan metabolism through inhibiting IL6/STAT3/VEGF signaling.

**Conclusions:** Collectively, our data demonstrate that miR-153 exerts anti-tumor activity in bladder cancer by targeting IDO1 expression. Future studies will investigate miR-153 as a novel therapeutic target for bladder cancer patients.

## Introduction

Bladder cancer is one of the most commonly occurring malignancies worldwide, accounting for an estimated 429,800 new cases and more than 165,000 cancer-related deaths in 2012, with an increased prevalence in males (1). Risk factors of bladder cancer include tobacco smoke, family cancer history, frequent bladder infections, and exposure to certain chemicals (2, 3). To date, ~75% of clinically diagnosed bladder cancers are non-muscle invasive and 25% involve bladder muscle (4). Treatment options for bladder cancer depend on tumor stage and include transurethral resection (TURBt), intravesical instillation, immunotherapy [Bacillus Calmette–Guérin (BCG)], and chemotherapy (5, 6). Compared to non-muscle invasive bladder cancer, tumors invading the muscle often have a poor prognosis (4). However, high rates of tumor recurrence are a clinical burden for all bladder cancer patients. Although we have obtained some useful results in study of the molecular mechanisms of bladder cancer (7, 8), further studies of bladder cancer etiology, molecular mechanisms, and pathogenesis could help identify novel strategies for early diagnosis and effective control of bladder cancer.

MicroRNA (miRNA or miR) is a class of single-stranded, non-coding RNA molecules that are ~18–22 nucleotides in length (9). MiRNAs function post-translationally to regulate expression of protein-coding genes through binding to the untranslated region (UTR; most at the 3′ end) of target mRNAs to promote either degradation of the targeting mRNAs, suppression of mRNA translation, or both. Thus, miRNAs negatively regulate the effect of targeting genes in cells (10). miRNAs can regulate cell growth, differentiation, migration, cell cycle, apoptosis, angiogenesis, and embryo development, whereas alteration of miRNAs could induce development of human diseases and carcinogenesis (9). To date, many studies have shown aberrant miRNA expression in human cancers, including bladder cancer (11–13). For example, a recent study revealed a urinary microRNA signature for accurate diagnosis of bladder cancer, independent of tumor stage and grade (14). Another recent study comprehensively analyzed the miRNA-mRNA regulatory network in gemcitabine-resistant bladder cancer cells (15), while a recent integrated analysis confirmed the usefulness of miRNAs as novel predictors of urologic cancer prognosis (16). Pardini et al. profiled different miRNA expression patterns to stratify bladder cancer subtypes (17). Moreover, Fujii et al. reviewed recent advancements in miRNA research with regards to smoking-related carcinogenesis to develop novel biomarkers and therapeutic strategies and better understand miRNA functions in cells (18). Robertson et al. performed a comprehensive analysis of 412 muscle-invasive bladder cancers using multiple TCGA datasets to identify expression of various mRNAs, long non-coding RNAs, and miRNAs for differential epithelial-mesenchymal transition (EMT) status of bladder cancer, carcinoma *in situ*, histology, and prognosis, as well as response of patients to different treatments (19). van Kampen et al. revealed the effects of a given miRNA to reverse tumor cell EMT and gene targeting (20).

miR-153 was reported to play an important role in the development of several types of human cancers. For example, a recent study showed that chromatin-modifying drugs upregulated miRNA-153 expression in glioblastoma cell lines, indicating that miR-153 is a tumor suppressor gene (21). miR-153 was downregulated in osteosarcoma tissues and expression of miR-153 suppressed osteosarcoma cell proliferation and invasion *in vitro* (22). miR-153 was also shown to be predictive of gastric cancer prognosis, and expression of miR-153 inhibited tumor cell migration and invasion capacity (23). Thus, in this study, we first assessed miR-153 expression in bladder cancer compared to adjacent normal tissue specimens and then investigated the underlying molecular mechanism of miR-153 mediated bladder cancer cell regulation. Our study provides new information regarding miR-153 as a potential tumor biomarker or novel therapeutic target for bladder cancer.

## Materials and Methods

### Patients and Tissue Specimens

This study collected normal and cancerous tissue specimens from 45 bladder cancer patients from Shanghai Tenth People's Hospital, Tongji University (Shanghai, China) between January 2017 and January 2018. These patients were histologically diagnosed with bladder cancer and did not receive any intravesical instillation or chemotherapy before surgery. Fresh tissue specimens were harvested during surgery and immediately snap-frozen in liquid nitrogen and stored at −80°C until use. This study was approved by the Ethics Committee of Shanghai Tenth People's Hospital and each patient provided informed consent before enrolling in the study. Clinicopathological data were retrieved from medical records, as shown in Table 1.

**Table 1:** Association of miR-153 expression with clinicopathological data from patients.

**Parameters**	**No. of patients**	**High expression**	**Low expression**	***P*-value**
Gender				1.00
Male	42	7	35	
Female	3	1	2	
Age (years)				0.39
≤ 65	19	4	15	
>65	26	4	22	
Histologic grade				1.00
High	41	7	34	
Low	4	1	3	
Tumor stage				0.031
I-II	20	6	11	
III-IV	25	2	26	
Tumor size (cm)				0.030
>3	23	2	27	
≤ 3	22	6	10	
Tumor Progress				0.009
Yes	27	1	26	
No	18	6	12	

### Reagents and Elisa Assay

Human recombinant interleukin 6 (IL-6; PeproTech, USA) was dissolved in trehalose at a concentration of 100 μg/mL and stored at −20°C. At the time of use, the final concentration of IL-6 was adjusted to 100 ng/mL in the appropriate medium. Human IL6 Elisa kit was purchased from Anogen-Yes Biotech Laboratories Ltd (EL10023, Ontario, Canada) and the concentration of IL6 in the cell supernatant was determined according to the manufacturer's instructions.

### Retrieval of the Cancer Genome Atlas (TCGA) Dataset

The Cancer Genome Atlas (TCGA) is a web-based database that attempts to map genomic variants of selected human cancers through large-scale genome sequencing of miRNA arrays and RNA-Seq. Currently, the TCGA contains 50 tumors and their subtypes. In this study, we downloaded the bladder cancer dataset (http://tcga-data.nci.nih.gov/tcga/) and assessed miR153 expression in 408 bladder cancer tissues and 19 normal tissues.

### Cell Lines, Culture, and Transfection

Human bladder cancer T24, UMUC3, 5637, and J82 cell lines and an immortalized human normal bladder epithelial cell line, SV-HUC-1, were obtained from the Chinese Academy of Sciences (Shanghai, China). T24, UMUC3, and 5637 cell lines were maintained in RPMI-1640 medium (Thermo Fisher Scientific, Inc., Waltham, MA, USA), J82 cells were cultured in Dulbecco's modified Eagle's medium (DMEM; Thermo Fisher Scientific, Inc.), and SV-HUC-1 cells were grown in F12K medium (Sigma-Aldrich, St. Louis, MO, USA) supplemented with 10% fetal calf serum (FCS; Thermo Fisher Scientific, Inc.) and 1% penicillin/streptomycin (Hyclone, Logan, UT, USA) in a humidified incubator containing 5% CO_2_ at 37°C.

To overexpress miR-153 in tumor cells, we purchased miR-153 mimics and the negative control from Genechem (Shanghai, China) into bladder cancer cell lines using.T24 and UMUC3 cells were seeded into 6-well plates in RPMI-1640 media supplemented with 10% FBS. At 70% confluence, the cells were transfected with Lipofectamine 3000 (Invitrogen; Thermo Fisher Scientific, Inc.) according to the manufacturer's instructions. The cells were harvested 48 h after transfection and subjected to analysis by qRT-PCR or western blotting. To knockdown IDO1 expression in tumor cells, we obtained IDO1 siRNA and negative control siRNA from Genepharma (Shanghai, China). We transfected the siRNAs into bladder cancer cell lines using Lipofectamine 3000 (Invitrogen). These transfected cell lines were then subjected to different assays. These sequences are shown in Supplementary Table 1.

### RNA Isolation and qRT-PCR

Total cellular RNA was isolated from tissue specimens and cell lines using a Trizol reagent (Invitrogen, Carlsbad, CA, USA) and reverse transcribed into cDNA using a cDNA synthesis kit (Takara, Dalian, China) according to the manufacturers' instructions. qPCR amplification was done using the SYBR Green PCR Kit (Takara) in the ABI Prism 7500 Sequence Detection System (Applied Biosystems, Foster City, CA, USA). The primers were show in Supplementary Table 1. U6 was used as the internal control for miR-153 mimics, and GAPDH was used as the internal control for IDO1. The PCR parameters were set for an initial cycle of 95°C for 2 min and 40 cycles at 95°C for 30 s, 57°C for 45 s, and 72°C for 45 s. The relative levels of each gene were analyzed using the 2^−ΔΔCq^ method. Experiments were done in duplicate and repeated three times.

### Western Blot

Total cellular protein was extracted from cells or tissues using radio immunoprecipitation assay buffer (RIPA buffer; Beyotime, Shanghai, China) and quantified.

The reaction mixture was incubated on ice for 30 min and centrifuged for 10 min at 12,000 g at 4°C. The supernatant was collected, and the protein concentration was estimated using a Pierce BCA protein assay kit (Thermo Fisher Scientific, Inc.) Proteins (30 μg per sample) were separated in 10% sodium dodecyl sulfate-polyacrylamide gel (SDS-PAGE) gels and transferred onto nitrocellulose (NC) membranes (Sigma-Aldrich). The membranes were blocked in 5% skim-milk solution in phosphate buffered saline (PBS) for 1 h at room temperature and then immunoblotted with a primary antibody at 4°C overnight. Primary antibodies included: IDO1 (1:1,000, ab211017, Abcam, USA), STAT3 (1:1,000, ab68153, Abcam), phosphorylated (p)-STAT3 (1:1,000, ab76315, Abcam), vascular endothelial growth factor (VEGF) (1:1,000, ab52917), E-cadherin (1:5,000, ab40772, Abcam), N-cadherin (1:5,000, ab76011, Abcam), and vimentin (1:5,000, ab92547, Abcam). β-actin (sc-47778, Santa Cruz Biotechnology, Santa Cruz, CA, USA) was used as an internal control. On the next day, the membranes were washed with PBS-Tween 20 (PBS-T) thrice and then incubated with fluorescence-conjugated secondary antibodies for 1 h at room temperature and then visualized with the Odyssey two-color infrared laser imaging system (LI-COR Biosciences, Lincoln, NE, USA).

### Cell Viability and Colony Formation Assays

Cell viability was measured using the Cell Counting Kit-8 (CCK-8; Dojindo Molecular Technologies, Inc., Kumamoto, Japan). In brief, transfected cells were seeded into 96-well plates at a density of 1 × 10^3^ cells/well and cultured for up to 3 days. After 6, 24, 36, or 72 h, we added 10 μl of the CCK-8 reagent into each well and further cultured the cells at 37°C for 2 h and then measured the absorbance rate on a microplate spectrophotometer (BioTek, Instruments, Inc., Winooski, VT, USA) at 450 nm.

For the cell colony formation assay, transfected cells were seeded in 6-well plates at 1 × 10^3^ /well and grown for 14 days with regular cell growth medium refreshment. At the end of each experiment, cells were washed three times with ice-cold PBS, fixed with 75% ethanol, and then stained with 0.1% crystal violet solution. Tumor cell colonies were counted from cell photographs that were imaged using an inverted microscope with a digital camera (Olympus Corporation, Tokyo, Japan).

### Tumor Cell Transwell Migration and Invasion Assay

Transwell chambers were obtained from Corning (Lowell, MA, USA). For the assay, 5 × 10^4^ transfected cells were seeded into the upper chamber of the Transwell in 200 μl medium without FCS, while the bottom chamber was filled with 600 μl culture medium containing 10% FCS. Cells were cultured for 16 h for the tumor cell migration assay or 24 h for invasion assay at 37°C. Cells that remained in the top surface of the Transwell filters were removed using a cotton swab, and the cells that migrated into or invaded the low surface of the filters were washed three times with iced-cold PBS, fixed with 70% ethanol for 30 min, stained with 0.5% crystal violet solution for 15 min, and counted under a light microscope (Olympus Corporation, Tokyo, Japan). For the cell invasion assay, the Transwell filters were pre-coated with Matrigel (BD Biosciences, Franklin Lakes, NJ, USA).

### Nude Mouse Xenograft Assay

The animal protocol in this study was approved by the Institutional Animal Care and Use Committee (IACUC) of Shanghai Tenth People's Hospital, Tongji University (Shanghai, China) and followed the regulatory animal care guidelines of the United State National Institute of Health (Bethesda, MD, USA). In brief, eight 4–5 week old male nude BALB/c mice were purchased from Shanghai SIPPR-Bk Lab Animal Co., Ltd. (Shanghai, China) and maintained in a specific pathogen-free (SPF) “barrier” facility and housed under controlled temperature and humidity with alternating 12 h light and dark cycles. Mice received SPF mouse chow and were allowed to drink sterile water *ad libitum*. Mice were subcutaneously injected with 1 x 10^6^ cells and tumor xenograft volume and size were measured weekly with a vernier caliper and calculated as follows: length × width^2^/2. After 40 days, we sacrificed the mice and collected all tumor xenografts for analysis.

### Tube Formation Assay

The capillary tube formation assay was used to assess the effect of miR-153 on HUVEC angiogenesis *in vitro*. In brief, Matrigel (12.5 mg/ml from BD Biosciences, Bedford, MA, USA) was thawed overnight at 4°C and 50 μl Matrigel was then added into each well of a 96-well plate and allowed to solidify at 37°C for 1 h. Then, 5,000 HUVECs were plated into each well and cultured in medium containing supernatants of miR-153 or control cultured cells for 6 h. Tube formation was observed under an inverted microscope (Olympus Corporation, Tokyo, Japan), photographed, counted, and summarized.

### Chicken Chorioallantoic Membrane (CAM) Assay

SPF fertilized eggs (Health-Tech Lab, Shandong, China) were incubated at 37°C for 5 days under constant humidity and a window on the shell was opened to expose the CAM. Next, 5 × 10^6^ cells were added onto the CAM and the eggs were incubated for 48 h to assess vascular branches and lengths, which were quantified using angiogenesis measurement software from KURABO (Osaka, Japan).

### Flow Cytometry

Apoptosis was measured using the Annexin V-FITC Apoptosis Kit (BD Biosciences, Erembodegem, Belgium). Transfected cells were harvested, washed twice with cold PBS and resuspended in Annexin V binding buffer. Thereafter, the cells were stained with fluorescein isothiocyanate (FITC) and propidium iodide (PI) for 15 min at 4° C in the dark. Apoptosis rate was measured using a BD FACS Calibur (Beckman Coulter, CA, USA).

CD34 expression on HUVECs was assayed using flow cytometry after co-culturing HUVECs with transfected bladder cells. In brief, after cell co-culture, HUVECs were collected and incubated on ice for 30 min in a fluorescence-activated cell-sorting buffer (BD Biosciences, Erembodegem, Belgium) containing a mouse anti-human CD34 antibody (#550761; BD Biosciences) and then analyzed using BD FACS Calibur. The percentage of positive cells was quantified using FlowJo software (BD Biosciences). The experiments were performed in duplicate and repeated three times.

### Dual Luciferase Reporter Assay

The target genes were predicted using bioinformatics analysis tools, including TargetScan (http://www.targetscan.org/vert_72/), ComiR (http://www.benoslab.pitt.edu/comir/) and miRANDA (http://www.microrna.org/).

To confirm the presence of miR-153 binding sites in the IDO1 3′-UTR, IDO1-wild-type 3′-UTR (IDO1-wt), and IDO1-mutant 3′-UTR (IDO1-mut) luciferase pmirGLO reporter vectors were constructed. Specifically, cells were seeded at a density of 1 × 10^5^ per well into 12-well plates and grown overnight and then co-transfected with either 100 nmol of the miR-153p mimics or the miR-NC plus 100 ng pmirGLO-30-UTR plasmid using Lipofectamine 3000 (Invitrogen) for 48 h. Next, total cellular protein was extracted and quantified using a routine laboratory protocol. Luciferase activity was measured using a dual luciferase reporter system (Promega Corporation, Madison, WI, USA) according to the manufacturer's protocol.

### Ultra-High-Performance Liquid Chromatography-Mass Spectrometry (UHPLC-MS) Analysis

Cells were seeded into 10 cm culture dishes and cultured for 24 h and then centrifuged at 10,000 g for 10 min at 4°C to collect the supernatant for metabolomics analysis. Cell supernatants were separated using the Agilent 1290 Infinity LC Ultra High-Performance Liquid Chromatography System (UHPLC) HILIC column at 25°C. The flow rate was 0.3 mL/min following an initial injection volume of 2 μL of each of sample. The conditions of the mobile phase included composition A with water, 25 mM ammonium acetate, plus 25 mM ammonia, while composition B included acetonitrile. The gradient elution was set at 0–1 min, 95% B; 1–14 min, B varied linearly from 95 to 65%; 14–16 min, B was changed linearly from 65 to 40%; 16–18 min, B was maintained at 40%; 18–18.1 min, B was changed linearly from 40 to 95%; and 18.1–23 min, B remained at 95%. The samples were then subjected to mass spectrometry using a Triple TOF 5600 mass spectrometer (AB SCIEX). Electrospray ionization (ESI) positive and negative ion modes were used for signaling detections according to the manufacturer's protocol.

### Statistical Analysis

All statistical analyses were performed using the SPSS software (version 22; SPSS, Inc., Chicago, IL, USA) or Graphpad (Version 5.0, GraphPad Prism Software Inc., San Diego, CA, USA). The continuous correction Chi-square test was used to assess the association between clinicopathological features and miR-153 expression, and the Student's *t*-test was performed to analyze differences between groups. A *P* < 0.05 was considered statistically significant.

## Results

### miR-153 Is Downregulated in Bladder Cancer Tissues and Cell Lines

**Figure 1:**
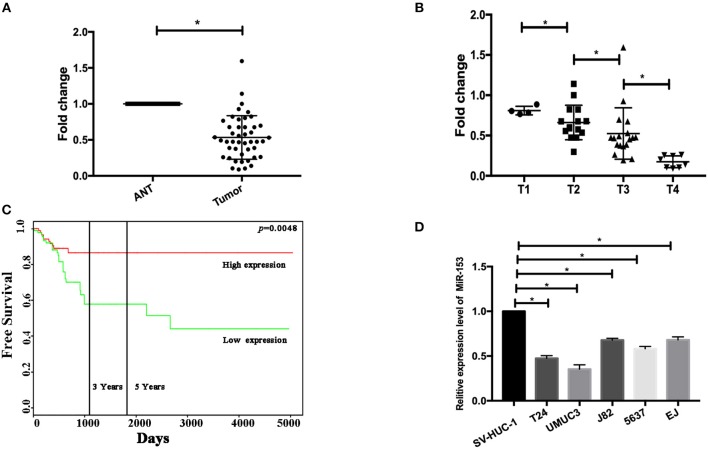
miR-153 is downregulated in bladder cancer tissues and cell lines. **(A)** qRT-PCR. miR-153 was detected in 45 pairs of bladder cancer (tumor) and adjacent normal tissues using qPCR (*P* < 0.05). **(B)** Association of miR-153 with clinicopathological features. Decreased miR-153 levels are associated with advanced tumor T stages. **(C)** Kaplan-Meier curve and log rank test stratified by miR-153 expression in TCGA dataset (http://tcga-data.nci.nih.gov/tcga/). **(D)** qRT-PCR. Various bladder cancer cell lines (T24, UMUC3, J82, 5637, and EJ) and an immortalized bladder epithelial cell line (SV-HUC-1) were grown and used for qPCR analysis (*P < 0.05).

In this study, we first measured miR-153 expression in bladder cancer tissue specimens and cell lines compared to normal samples and found that miR-153 expression was significantly downregulated in 45 pairs of bladder cancer compared to adjacent normal tissues (ANT; *P* < 0.05; Figure 1A). The reduced miR-153 expression was associated with advanced tumor stage (Figure 1B). However, since our patients were enrolled between 2017 and 2018 with a very short follow-up period, we used the TCGA dataset to determine the association of miR-153 levels with bladder cancer prognosis. Our Kaplan-Meier curves and log rank test showed that reduced miR-153 expression was associated with worse overall survival of patients (Figure 1C). Consistently, miR-153 expression was also lower in different bladder cancer cell lines T24, UMUC3, 5637, and J82, compared to expression in the normal bladder epithelial cell line SV-HUC-1. Among these tumor cell lines, T24 and UMUC3 cells expressed the lowest level of miR-153 (Figure 1D) and were therefore used for overexpression of miR-153 in the following experiments.

### miR-153 Inhibits Bladder Cancer Growth *in vitro* and *in vivo* by Promoting Tumor Cell Apoptosis

**Figure 2:**
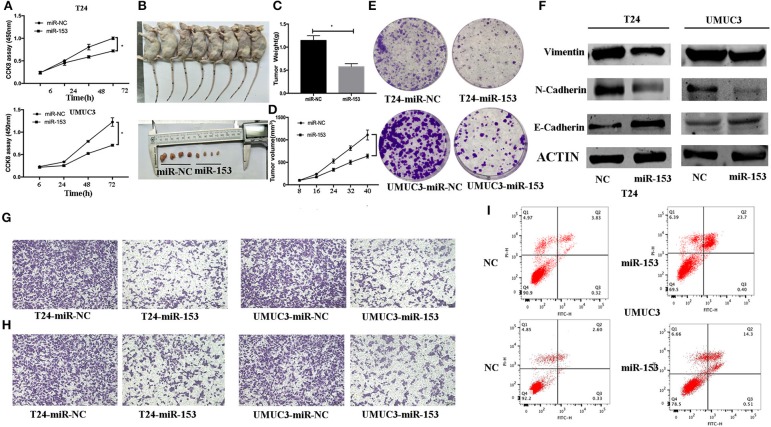
miR-153 inhibits bladder cancer growth *in vitro* and *in vivo* by promoting tumor cell apoptosis, migration, invasion, and EMT. **(A)** Cell viability CCK-8 assay. T24 and UMUC3 cells were transfected with miR-153 mimics or negative control and then subjected to the CCK-8 assay. **(B)** Nude mouse xenograft assay. Stably miR-153 expressing mimics or negative control bladder cancer cells were subcutaneously injected into nude mice and monitored for 40 days for tumor cell xenograft formation and growth. **(C)** Tumor cell xenograft growth curves. **(D)** Tumor cell xenograft weight. **(E)** Colony formation assay. T24 and UMUC3 cells were transfected with miR-153 mimics or negative control and then subjected to tumor cell colony formation assay (x 200). **(F)** Western blot. Expression levels of EMT-associated markers in miR-153 mimics or negative control transfected T24 and UMUC3 cells were evaluated by using Western blot analysis. **(G)** Transwell tumor cell migration assay. **(H)** Transwell tumor cell invasion assay. **(I)** Flow cytometric Annexin V-PI double staining assay. *P < 0.05.

Next, we assessed the effects of miR-153 expression on bladder cancer cell proliferation. Transfection of miR-153 mimics into T24 and UMUC3 cells significantly increased miR-153 expression compared to miR-NC control cells (Figure 2A). We measured the effect of miR-1543 overexpression on tumor cell growth and found that miR-153 overexpression significantly reduced viability and colony formation in T24 and UMUC3 cells (Figures 2A,E). Consistently, our nude mouse experiments revealed that growth of tumor cell xenografts after miR-153 overexpression was also suppressed compared to the miR-NC groups (Figures 2B–D). Furthermore, the apoptosis rate of miR-153-expressing tumor cells was upregulated compared to the negative controls (Figure 2I).

### miR-153 Inhibits Bladder Cancer Cell Migration, Invasion, and EMT

We evaluated the effect of miR-153 on bladder cancer cell migration and invasion capacity. Our data showed that miR-153 mimics significantly reduced T24 and UMUC3 cell migration and invasion compared to the negative controls (Figures 2G,H). Furthermore, during tumor cell EMT, E-cadherin expression (epithelial cell marker) was reduced, whereas N-cadherin and vimentin expression (mesenchymal cell markers)were increased induced (24). Our *in vitro* data showed that miR-153 overexpression reduced N-cadherin and vimentin expression, but increased E-cadherin expression (Figure 2F).

### miR-153 Inhibition of Bladder Cancer Angiogenesis *in vivo* and *in vitro*

**Figure 3:**
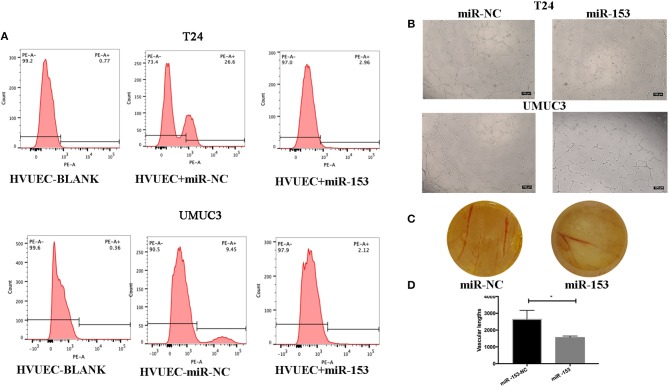
miR-153 inhibits bladder cancer angiogenesis *in vitro* and *in vivo*. **(A)** Flow cytometric assay. CD34 expression on HVUECs was assessed using flow cytometry in after co-culture with miR-153 or negative control transfected T24 and UMUC3 cells. **(B)** HUVEC tube formation assay. HUVECs were co-cultured with T24 and UMUC3 cells using Transwells and then subjected to the capillary-like tube formation assay (x100). **(C)** The CAM angiogenesis assay. miR-153 or negative control transfected T24 cells were added on to CAM to induce angiogenesis for 48 h to induce formation of vascular branches (x 10). **(D)** The vascular length are measured (*p < 0.05).

We also assessed the effect of miR-153 expression on tumor angiogenesis *in vitro*. We found that miR-153 overexpression altered bladder cancer angiogenesis. Specifically, co-culture of stably miR-153 expressing bladder cancer cells with HVUECs using Transwell chambers (0.4 μm) reduced the ability of HVUECs to form tube-like structures *in vitro* (Figure 3B). At the protein level, CD34 expression on HVUECs was significantly reduced after co-culturing with miR-153-expressing bladder cancer cells compared to control HVUECs (Figure 3A). Furthermore, the CAM angiogenesis assay confirmed the tube formation data, showing that miR-153 expression reduced *in vivo* angiogenesis (Figures 3C,D).

### miR-153 Targets IDO1 Expression and Inhibits Bladder Cancer Cell Tryptophan Metabolism

**Figure 4:**
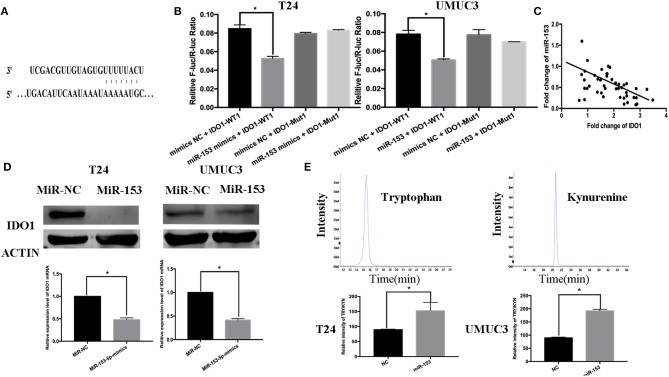
IDO1 is a direct miR-153 target. **(A)** Bioinformatical analysis predicted that IDO1 is a direct miR-153 target. **(B)** Luciferase reporter assay. miR-153 or miR-NC-transfected bladder cancer cells were co-transfected with a luciferase reporter plasmid (WT or MUT 3 -UTR IDO1 cDNA) and then subjected to protein extraction and luciferase reporter assay. **(C)** miR-153 is negatively correlated with the expression of IDO1 in cancer tissues by qRT-PCR **(D)** qRT-PCR and Western blot. miR-153 or negative control transfected T24 and UMUC3 cells were grown and subjected to qRT-PCR and Western blot. **(E)** UHPLC-MS. miR-153 or negative control transfected T24 and UMUC3 cells were grown and the supernatants were subjected to UHPLC-MS; comparison of the tryptophan ratio to kynurenine. *P < 0.05.

Thus far, we revealed the anti-tumor activity of miR-153 in bladder cancer cells *in vitro* and *in vivo*; however, the biological function of miRNAs is to regulate expression of their targeting genes. In this regard, we performed bioinformatical analysis to predict the target genes of miR-153. We found that IDO1 was a miR-153 target. Specifically, miR-153 is predicted to bind to the 3′-untranslated region (UTR) of IDO1 cDNA (Figure 4A). To confirm this, we performed a luciferase reporter assay and found that miR-153 inhibited luciferase activity of the wild type 3′-UTR IDO1 cDNA, but not of the mutated cDNA (Figure 4B). Furthermore, we found an inverse association of IDO1 expression with miR-153 expression in 45 bladder cancer tissue samples (Figure 4C). In addition, miR-153 overexpression significantly downregulated levels of IDO1 mRNA and protein in T24 and UMUC3 cells (Figure 4D). IDO1 is the key enzyme in tryptophan metabolism and we therefore assessed whether tryptophan metabolism was altered using UHPLC-MS of the supernatants of miR-153-expressing and negative control T24 and UMUC3 cells (Figure 4E). We found that miR-153 expression suppressed tryptophan metabolism, and the ratio of tryptophan/kynurenine (TRY/KYN) was significantly higher in miR-153 expressing tumor cells compared to the control cells.

### IDO1 Suppression and miR-153 Overexpression Have Parallel Effects in Bladder Cancer Cells *in vitro*

**Figure 5:**
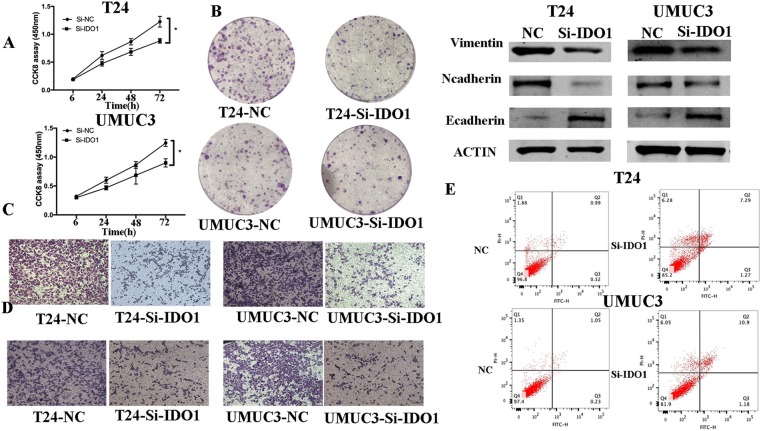
IDO1 knockdown inhibits bladder cancer cell proliferation, migration, and invasion, and induced apoptosis and modulation of EMT markers. **(A)** Cell viability CCK-8assay. T24 and UMUC3 cells were transfected with IDO1 or negative control siRNA and then subjected to the CCK-8 assay. **(B)** Colony formation assay. T24 and UMUC3 cells were transfected with IDO1 or negative control siRNA and then subjected to colony formation (x 200) and Transwell assays. **(C)** Western blot. Levels of the EMT-associated markers were analyzed in T24 and UMUC3 cells after IDO1 knockdown by using Western blot. **(D)** Transwell migration assay. **(E)** Transwell invasion assay. **(E)** Flow cytometric Annexin V-PI double staining assay in T24 and UMUC3 cells after knockdown of IDO1. *P < 0.05.

**Figure 6:**
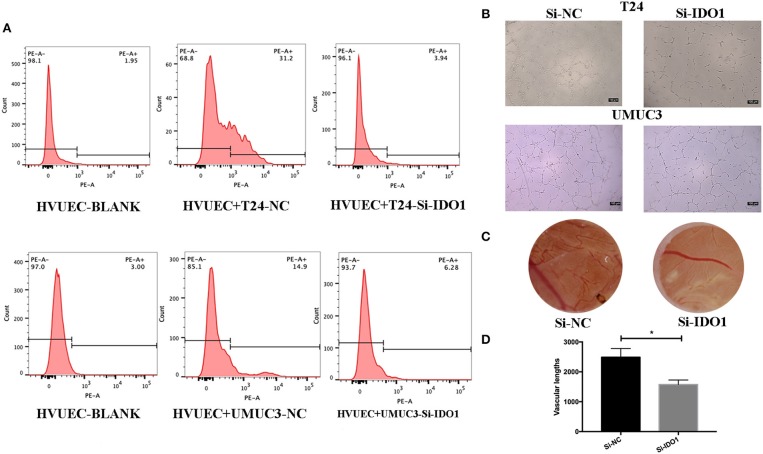
IDO1 knockdown inhibits angiogenesis. **(A)** Flow cytometric assay. HUVECs were co-cultured with IDO-siRNA or negative control siRNA-transfected T24 and UMUC3 cells and then subjected to flow cytometric analysis of CD34 level on HUVECs. **(B)** HUVEC tube formation assay. The same cultured HUVECs were subjected to the tube formation assay (x100). **(C,D)** The CAM assays. The siRNA-transfected T24 cells were added onto CAM to modulate angiogenesis for 48 h (x 10). The branches and length are measured (*p < 0.05).

To further confirm the biological function of IDO1 in bladder cancer cells, we knocked down IDO1 expression in T24 and UMUC3 cells using si-IDO1 and found that si-IDO1 significantly reduced levels of IDO1 mRNA and protein in T24 and UMUC3 cells compared to the negative controls. Knockdown of IDO1 expression reduced tumor cell growth, colony formation, migration, and invasion, but induced tumor cell apoptosis. Inhibition of IDO1 expression also modulated expression of EMT markers (Figure 5) and HUVEC angiogenesis. These findings are consistent with miR-153 overexpression in bladder cancer cells, suggesting that the effects of miR-153 on bladder cancer cells are mediated through targeting IDO1 expression (Figure 6).

### miR-153 Targets IDO1 and Modulates Angiogenesis Through IL6/STAT3/VEGF Signaling

**Figure 7:**
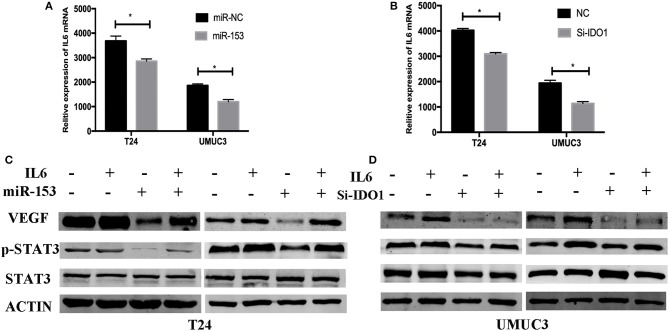
miR-153 targets IDO1 and modulates angiogenesis through IL6/STAT3/VEGF signaling. **(A,B)** ELISA. IL_6 expression in T24 and UMUC3 cells (overexpression of miR-153 or knockdown of IDO1 and their respective negative controls) were analyzed using ELISA. **(C,D)** Western blot. T24 and UMUC3 cells (overexpression of miR-153 or knockdown of IDO1 and their respective negative controls) were pretreated with 100 ng/ml of IL-6 for 48 h and then subjected to Western blot analysis of STAT3, p-STAT3, and VEGF.

**Figure 8:**
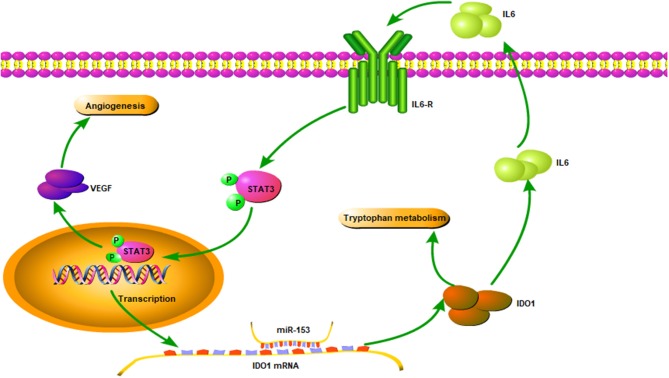
Illustration of the miR-153 gene pathway in bladder cancer cells. miR-153 expression is lost in bladder cancer, which decreases IDO1 expression and subsequent activity of the IL6/STAT3/VEGF signaling pathway in bladder cancer cells.

To explore the underlying molecular mechanism of miR-153 in bladder cancer, we measured interleukin-6 (IL6) expression in supernatants of miR-153-overexpressing or IDO1 knocked down bladder cancer cells compared to the negative control cells. We found that, IL-6 expression was significantly suppressed in the experimental groups compared to the control groups, while phosphorylated STAT3 and VEGF were also significantly decreased in miR-153-overexpressing or IDO1 knocked down bladder cancer cells (Figure 7). However, addition of exogenous IL-6 reversed levels of both phosphorylated STAT3 and VEGF expression (Figure 7). Thus, we speculate that the role of miR-153 in bladder cancer angiogenesis is to target IDO1 expression and regulate IL6/STAT3/VEGF signaling (Figure 8).

## Discussion

The role of miRNAs in cancer development and progression has recently received increasing attention. In bladder cancer, different miRNAs have been shown to be altered and associated with tumor development and progression. These miRNAs could be useful biomarkers for early detection, prediction of treatment response, subtyping, and prognosis of bladder cancer. In our current study, we measured miR-153 expression in bladder cancer compared to adjacent normal tissue specimens and then investigated the underlying molecular mechanisms of miR-153 on regulating bladder cancer cells. We found that miR-153 expression was reduced in bladder cancer tissues and cell lines, which was associated with advanced tumor stage and poor overall survival. Restoring miR-153 expression in bladder cancer cell lines inhibited tumor cell growth, migration, invasion, and EMT *in vitro* and tumor xenograft growth *in vivo*, while miR-153 expression also suppressed HUVEC and CAM angiogenesis. miR-153 targeted IDO1 expression and inhibited bladder cancer cell tryptophan metabolism through inactivating the IL6/STAT3/VEGF signaling pathway. In conclusion, our current study demonstrates that miR-153 exerts its anti-tumor activity in bladder cancer by targeting IDO1 expression.

Altered miR-153 expression and anti-tumor activity of miR-153 in bladder cancer have not been previously reported and are therefore novel findings. However, the role of miR-153 has been described in other human cancers. Xu reported miRNA-153 tumor suppressor activity in glioblastoma (21), while miR-153 was downregulated in osteosarcoma tissues (22), and loss of miR-153 expression was associated with poor gastric cancer prognosis (23). Moreover, miR-153 was downregulated in lung cancer and miR-153 expression inhibited lung cancer cell proliferation and migration, but promoted tumor cell apoptosis (25). Our current study further supports these published findings in human malignancies. However, Zhang et al. showed that miR-153 was highly expressed in advanced colorectal cancer and miR-153 promoted colorectal cancer cell invasion and cisplatin-resistance (26). Wu et al. reported that miR-153 was up-regulated in prostate cancer and miR-153 expression induced tumor cell proliferation and migration (27). These studies suggest that the role of miR-153 differs depending on the type and location of the cancer. Thus, further study of miR-153 in bladder cancer is needed to confirm miR-153's anti-tumor activity.

In our current study, we also demonstrated that the effect of miR-153 on inhibiting bladder cancer-induced HUVEC angiogenesis *in vitro* and *in vivo*. At the molecular level, miR-153 suppressed IDO1 expression by targeting IDO1 3′-UTR. Indeed, IDO1 is a rate-limiting enzyme in tryptophan metabolism (28). The tryptophan catabolic pathway plays an important role in tumor cell escape (29, 30). Moreover, IDO1 has non-immune functions, such as angiogenesis. Su et al. reported that Erianin exerted it anti-angiogenic ability by suppressing HUVEC tube formation through downregulating IDO1 expression (31). Wei et al. also showed that IDO1 expression in breast cancer was associated with tumor microvessel density and poor overall survival of patients (32). In addition, an IDO1 knockout mouse lung metastasis model showed that pulmonary vessel density was significantly reduced (33). These studies demonstrated the role of IDO1 in angiogenesis and our current study is the first to report that miR-153 inhibits IDO1 expression to suppress tryptophan metabolism and angiogenesis. Our current study also revealed that both miR-153 expression and IDO1 knockdown reduced CD34 expression and inhibited HUVEC angiogenesis. This conclusion was also verified in the CAM assay.

Our current study further demonstrated that miR-153's anti-tumor activity in bladder cancer cells was mediated by targeting IDO1, which in turn inactivated the IL6/STAT3/VEGF signaling pathway. Several previous studies showed that STAT3 expression enhanced tumor angiogenesis (34–36). STAT3 can be activated by various cytokines (such as IL6 and non-IL6 family members), which bind to the cell surface receptors resulting in phosphorylation of STAT3 downstream molecules, such as VEGF (37). Indeed, our current data showed that high IDO1 expression promoted IL-6 secretion in bladder cancer cells. Vacher et al. reported that IDO1 induced secretion of IL1B, IL6, IL8, and CXCR4 in breast cancer (38). IL6 expression also promoted tryptophan metabolism to constitute a positive feedback loop (39). In the current study, we found that miR-153 expression reduced IL6 secretion in bladder cancer cells, thereby inhibiting STAT3 signaling and VEGF expression. However, addition of exogenous IL6 to tumor cell culture can restore STAT3 signal transduction and increase VEGF expression. This finding demonstrates that miR-153's anti-angiogenesis activity is regulated through the IL6-mediated STAT3 signaling and targeting of IDO1.

To our knowledge, we are the first to demonstrate that miRNAs can regulate metabolomics in bladder cancer. Specifically, we found that miR-153 expression significantly affected tryptophan metabolism by targeting IDO1 expression and showed a significant association between tryptophan metabolism and tumor growth. In a previous study, Kesarwani et al. showed that the level of tryptophan detected by mass spectrometry was significantly correlated with the efficacy of glioblastoma radiotherapy, and a decrease in tryptophan levels inhibited the reactivation of immune checkpoints (40). Therefore, our future study will determine if there is an association of tryptophan metabolism and miR-153 expression in patient bladder cancer samples.

## Conclusions

In conclusion, our current study demonstrated that miR-153 is significantly downregulated in bladder cancer tissues and cell lines. Overexpression of miR-153 inhibited bladder cell proliferation, migration, invasion, *in vitro* angiogenesis, and *in vivo* tumor cell xenograft formation and growth. Furthermore, we found that IDO1 is a direct target of miR-153, which mediated miR-153 anti-tumor activity in bladder cancer via inactivating the IL6/STAT3/VEGF pathway. These data suggest that miR-153 may be a novel therapeutic target for bladder cancer and further study could provide more novel insights into the mechanisms of bladder cancer progression and metastasis.

## Ethics Statement

This study was approved by the Ethics Committee of Shanghai Tenth People's Hospital. All subjects gave written informed consent in accordance with the Declaration of Helsinki.

## Author Contributions

WZ wrote the manuscript and DS generated the figures. ZZ, YG, JZ, YW, LW, RW, and SM contributed to the acquisition of clinical and experimental data. YH and XY contributed to editing the manuscript. All authors read and approved the final manuscript.

### Conflict of Interest Statement

The authors declare that the research was conducted in the absence of any commercial or financial relationships that could be construed as a potential conflict of interest.
