# Corrigendum: MicroRNA-153 decreases tryptophan catabolism and inhibits angiogenesis in bladder cancer by targeting indoleamine 2,3-dioxygenase 1

**DOI:** 10.3389/fonc.2023.1208728

**Published:** 2023-05-22

**Authors:** Wentao Zhang, Shiyu Mao, Donghui Shi, Junfeng Zhang, Ziwei Zhang, Yadong Guo, Yuan Wu, Ruiliang Wang, Longsheng Wang, Yong Huang, Xudong Yao

**Affiliations:** ^1^ Department of Urology, Shanghai Tenth People’s Hospital, Tongji University, Shanghai, China; ^2^ Anhui Medical University, Shanghai Clinical College, Hefei, China; ^3^ Department of Urology, Suzhou Wuzhong People’s Hospital, Wuzhong, China; ^4^ Department of Urology, The First Affliated Hospital of Inner Mongolia Medical University, Hohhot, China

**Keywords:** bladder cancer, miR-153, tryptophan catabolism, angiogenesis, indoleamine 2, 3-dioxygenase 1

In the published article, there was a there was an error in **Figures 2**, **5** as published. We mistakenly used the same image of clone formation in **Figure 2E** (T24-miR-153) and **Figure 5B** (UMUC3-Si-IDO1) due to layer overlays. Furthermore, in the transwell section of **Figure 2**, we found a minor error in the UMUC3-miR-153(G) and T24-miR-NC(H) due to incorrect use of images. We repeated these experiments and corrected the results. The corrected **Figures 2**, **5** appear below.

**Figure 2:**
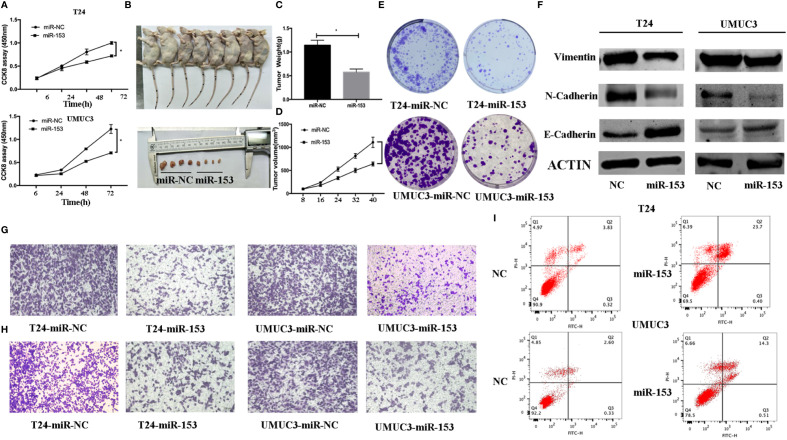
miR-153 inhibits bladder cancer growth *in vitro* and *in vivo* by promoting tumor cell apoptosis, migration, invasion, and EMT. **(A)** Cell viability CCK-8 assay. T24 and UMUC3 cells were transfected with miR-153 mimics or negative control and then subjected to the CCK-8 assay. **(B)** Nude mouse xenograft assay. Stably miR-153 expressing mimics or negative control bladder cancer cells were subcutaneously injected into nude mice and monitored for 40 days for tumor cell xenograft formation and growth. **(C)** Tumor cell xenograft growth curves. **(D)** Tumor cell xenograft weight. **(E)** Colony formation assay. T24 and UMUC3 cells were transfected with miR-153 mimics or negative control and then subjected to tumor cell colony formation assay (x 200). **(F)** Western blot. Expression levels of EMT-associated markers in miR-153 mimics or negative control transfected T24 and UMUC3 cells were evaluated by using Western blot analysis. **(G)** Transwell tumor cell migration assay. **(H)** Transwell tumor cell invasion assay. **(I)** Flow cytometric Annexin V-PI double staining assay. *P < 0.05.

**Figure 5:**
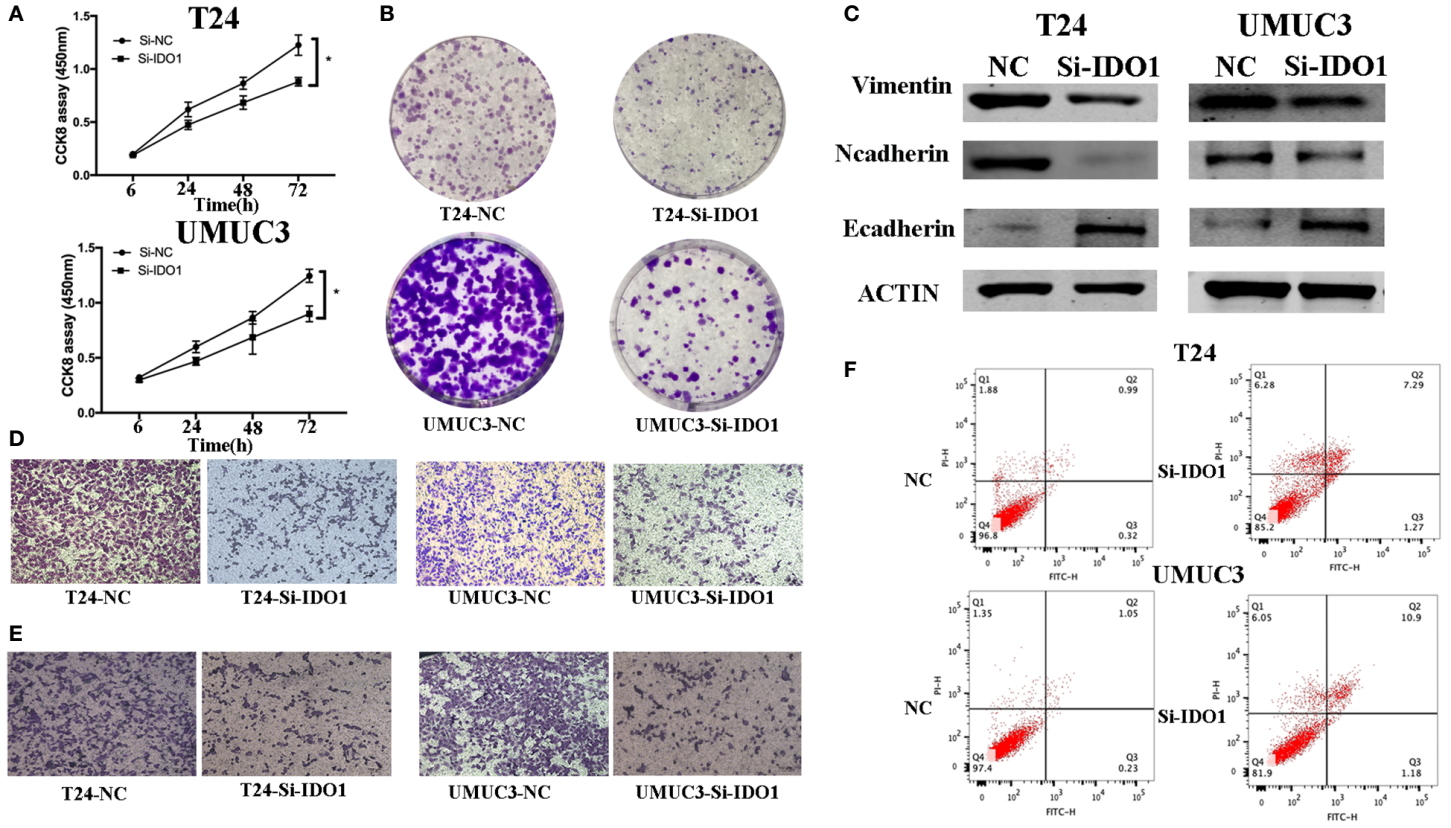
IDO1 knockdown inhibits bladder cancer cell proliferation, migration, and invasion, and induced apoptosis and modulation of EMT markers. **(A)** Cell viability CCK-8assay. T24 and UMUC3 cells were transfected with IDO1 or negative control siRNA and then subjected to the CCK-8 assay. **(B)** Colony formation assay. T24 and UMUC3 cells were transfected with IDO1 or negative control siRNA and then subjected to colony formation (x 200) and Transwell assays. **(C)** Western blot. Levels of the EMT-associated markers were analyzed in T24 and UMUC3 cells after IDO1 knockdown by using Western blot. **(D)** Transwell migration assay. **(E)** Transwell invasion assay. **(F)** Flow cytometric Annexin V-PI double staining assay in T24 and UMUC3 cells after knockdown of IDO1. *P < 0.05.

There was also an error in **Figure 7**. We missed labeling the names of T24 and UMUC3 cell lines in panels C and D. The corrected **Figure 7** appears below.

**Figure 7:**
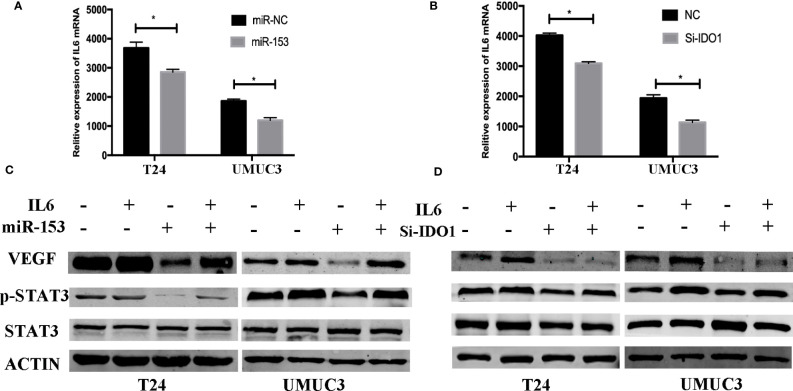
miR-153 targets IDO1 and modulates angiogenesis through IL6/STAT3/VEGF signaling. **(A, B)** ELISA. IL_6 expression in T24 and UMUC3 cells (overexpression of miR-153 or knockdown of IDO1 and their respective negative controls) were analyzed using ELISA. **(C, D)** Western blot. T24 and UMUC3 cells (overexpression of miR-153 or knockdown of IDO1 and their respective negative controls) were pretreated with 100 ng/ml of IL-6 for 48 h and then subjected to Western blot analysis of STAT3, p-STAT3, and VEGF. *p<0.05.

The authors apologize for this error and state that this does not change the scientific conclusions of the article in any way. The original article has been updated.

