# Supplementary material for: MicroRNA-153 Decreases Tryptophan Catabolism and Inhibits Angiogenesis in Bladder Cancer by Targeting Indoleamine 2,3-Dioxygenase 1
Source: Front Oncol. 2019 Jul 10;9:619. doi: 10.3389/fonc.2019.00619 (PMC6636202; doi:10.3389/fonc.2019.00619)
Supplement: Supplementary file 1 [file Table_1.DOCX]

| miR-153-mimics | 5'-UCAUUUUUGUAUGUUGCAGCU-3′ | sense |
| --- | --- | --- |
|  | 5'-CUGCAACAUCACAAAAAUGAUU-3′ | antisense |
| miR-153-NC | 5'-UCACAACCUCCUAGAAAGAGUAGA-3' | sense |
|  | 5’-UACUCUUUCUAGGAGGUUGUGAUU-3' | antisense |
| si-IDO1 | 5'-GAACGGGACA-CUUUGCUAA-3′ | sense |
|  | 5'-UUAGCAAAGUGUCCC-GUUC-3′ | antisense |
| si-NC | 5'-UUCUCCGAACGUGUCACGUTT-3′ | sense |
|  | 5'-ACGUGA-CACGUUCGGAGAATT-3′ | antisense |
| Primers |  |  |
| miR-153 | 5'-acgcgcgcgTCATTTTTGTGATGTT-3' | forward |
|  | 5'-ATCCAGTGCAGGGTCCGAGG-3' | reverse |
| U6 | 5'-TGCGGGTGCTCGCTTCGCAGC-3' | forward |
|  | 5'-CCAGTGCAGGGTCCGAGGT-3' | reverse |
| IDO 1 | 5'-GCTTGCAGGAATCAGGATGT-3' | forward |
|  | 5'-GGCAAAGGTCATGGAGATGT3' | reverse |
| GAPDH | 5'-ATGTCGTGGAGTCTACT GGC-3' | forward |
|  | 5'-TGACCTTGCCCACAGCCTTG-3' | reverse |

Supplementary Table1 This table lists the sequences of miR-153mimics and NC, the sequences of IDO1 SiRNA and NC used in this study, and primers for qRT-PCR .
